# REALMS: Resilient exploration and lunar mapping system

**DOI:** 10.3389/frobt.2023.1127496

**Published:** 2023-03-30

**Authors:** D. van der Meer, L. Chovet, A. Bera, A. Richard, Pedro Jesus Sánchez Cuevas, J. R. Sánchez-Ibáñez, M. Olivares-Mendez

**Affiliations:** ^1^ Space Robotics (SpaceR) Research Group, Interdisciplinary Centre for Security, Reliability and Trust (SnT), University of Luxembourg, Luxembourg City, Luxembourg; ^2^ Advanced Centre for Aerospace Technologies (CATEC), Seville, Spain; ^3^ Guidance Navigation and Control Department, Airbus Defence and Space Ltd., Stevenage, United Kingdom

**Keywords:** resilience, multi-master, delay invariant, mapping, VSLAM, lunar, exploration

## Abstract

Space resource utilisation is opening a new space era. The scientific proof of the presence of water ice on the south pole of the Moon, the recent advances in oxygen extraction from lunar regolith, and its use as a material to build shelters are positioning the Moon, again, at the centre of important space programs. These worldwide programs, led by ARTEMIS, expect robotics to be the disrupting technology enabling humankind’s next giant leap. However, Moon robots require a high level of autonomy to perform lunar exploration tasks more efficiently without being constantly controlled from Earth. Furthermore, having more than one robotic system will increase the resilience and robustness of the global system, improving its success rate, as well as providing additional redundancy. This paper introduces the Resilient Exploration and Lunar Mapping System, developed with a scalable architecture for semi-autonomous lunar mapping*.* It leverages Visual Simultaneous Localization and Mapping techniques on multiple rovers to map large lunar environments. Several resilience mechanisms are implemented, such as two-agent redundancy, delay invariant communications, a multi-master architecture different control modes. This study presents the experimental results of REALMS with two robots and its potential to be scaled to a larger number of robots, increasing the map coverage and system redundancy. The system’s performance is verified and validated in a lunar analogue facility, and a larger lunar environment during the European Space Agency (ESA)-European Space Resources Innovation Centre Space Resources Challenge. The results of the different experiments show the efficiency of REALMS and the benefits of using semi-autonomous systems.

## 1 Introduction

In recent years, space resources utilisation has become increasingly interesting from an economic and scientific perspective. The Moon contains valuable resources for *In-Situ* Resources Utilisation (ISRU) (Crawford, 2014). The most important resources are regolith for use as construction material, and water ice to generate rocket propellant and oxygen for life sustainability. Given the growing interest, countries are starting to establish legal frameworks (Pretto et al., 2021; Smith, 2021), allowing for more progress and innovation in ISRU. Luxembourg aims at becoming the leading country in space resources activities (Bloomberg, 2021), establishing a legal framework (Luxembourg Space Agency, 2021) and the European Space Resources Innovation Centre (ESRIC) in coordination with the European Space Agency (ESA) [Naujokaitytė, (2020)].

**FIGURE 1:**
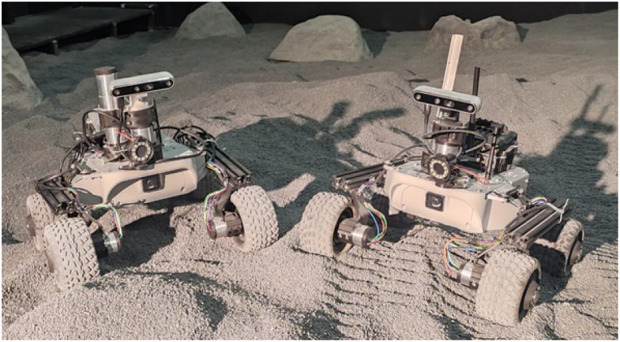
REALMS Leo rovers in the LunaLab.

The NASA Artemis program (National Aeronautics and Space Administration, 2022a) is leading a set of missions to find water ice on the lunar surface and perform ISRU, allowing astronauts to stay on the Moon for a long time. NASA plans to have the Volatiles Investigating Polar Exploration Rover (VIPER) exploring some Permanent Shadowed Regions (PSR) in the south pole to study the presence of water ice and to extract samples from them (National Aeronautics and Space Administration, 2022b). The pre-mission planning is based on the maps generated by using data from the Lunar Reconnaissance Orbiter (LRO). However, in the best cases, the map resolution in the south pole is half of the resolution of the maps from the equatorial regions (Delgado-Centeno et al., 2021). In addition, the south pole regions have large shadows generated by boulders and low incident angles of Sun rays that hide potentially hazardous areas. The rover could collide with non-detected small boulders, or the low temperatures in the shadowed regions could damage the robot’s electronics. Therefore, the success of this mission will strongly depend on the navigation sub-system of the VIPER rover to generate reliable mapping and localisation estimation in long traverses. Inspired by the VIPER mission, and to drive the innovation in technologies for space resources detection and prospecting, ESRIC and ESA have launched the Space Resources Challenge (ESA - European Space Agency, 2021). This is a lunar prospecting challenge, where each participating team has to explore and map a lunar analogue facility and analyse specific rocks within a limited time. The facility includes boulders, slopes, and low incident angle illumination to replicate the visual appearance of the lunar south pole. The facility’s communication system simulates Earth-Moon-Earth communication with five seconds delay, limited bandwidth, and connection losses. In this work, we present our REALMS approach that leads us to be qualified for the final round of this Space Resources Challenge. As part of the LUVMI-XR consortium, REALMS represents the scouting part of the mission, while the scientific analysis is performed by the LUVMI-X rover. This paper shows the REALMS system and performance of the scouting team formed by two Leo Rovers (Kell Ideas, 2021) (Figure 1).

A Multi-Robot System (MRS) provides increased coverage and improves the efficiency of specific tasks by executing them in parallel. As a result, the robotic system consists of multiple robots dedicated to a single task, distributing the mission risk across multiple agents and potentially reducing overall mission costs. In a mission focused on exploration and prospecting, MRS are one of the most interesting solutions, especially when mapping and analysing large surfaces in a short amount of time.

This paper proposes our REALMS, a multi-robot, scalable and resilient solution for lunar exploration and prospecting, adaptable to homogeneous and heterogeneous rovers. We summarise the contributions as stated below.

• We establish the communication between the robots and the ground stations to a platform independent protocol to make the system robust to Earth-Moon-Earth communication delay.
• We provide a solution to operate multiple rovers in semi-autonomous and teleoperated modes, which increases the efficiency and reliability.
• We integrate a Visual Simultaneous Localisation and Mapping (vSLAM) solution for a lunar environment.
• We perform the Verification and Validation (V&V) of REALMS with real rovers in two different lunar analogue facilities for short and long traverses within the context of the ESA-ESRIC Space Resources Challenge.
• We present guidelines for using ROS and multi-robot systems in a unique and hybrid approach to overcome problems generated in lunar environments with Earth-Moon-Earth communication*,* such as software timeouts that prevent robots from connecting to the ROS Master.

## 2 Related work

Traditional planetary space missions led by space agencies operated single multipurpose robots equipped with several sensors, actuators, and complex algorithms. Their primary goal was to collect as much data as possible about different minerals, rather than to explore large areas. Some examples were Lunokhod 2 (National Aeronautics and Space Administration, 2018) and Yutu-2 (Ma et al., 2020) for the Moon, and Sojourner (Heuseler, 1998) and Curiosity (Lakdawalla, 2018) for Mars. The twin rovers Spirit and Opportunity (Arvidson, 2011) were sent to different locations to perform non-coordinated tasks, hence not working together. A similar case were the most recent missions, Perseverance and Ingenuity, in which both robots acted as independent systems (National Aeronautics and Space Administration, 2021a). All these rovers were developed with a high level of redundancy and state-of-the-art sensors. Nonetheless, their missions were strongly constrained by potential mobility issues. Any movement had to be planned precisely. For instance, Perseverance had a maximum speed of 0.042 m/s (National Aeronautics and Space Administration, 2021b) to reduce potential risks. This implied a small-scale coverage, making any exploration mission long and requiring the full supervision of an operator. The coming years are expected to see the first private rover missions on the Moon (Lunar Outpost, 2021; ispace, 2021). Limited budgets for these missions will seek for efficiency and resilience. After an initial test mission, there will be more missions to perform exploration and prospecting using MRS.

The application of MRS had already been extensively studied in many fields such as agriculture (et al., 2021) or search and rescue (Yan et al., 2013). The work described in (Parker, 2008) distinguished four main architectures for MRS: First, a centralised approach to coordinate the fleet from one main computer, assuring a simpler robot design with lower computational requirements. However, this made the entire fleet dependent on the main computer, causing it to be fault intolerant, as discussed in (Caloud et al., 1990). Second, a hierarchical architecture in which each robot is either a part of a small fleet or a leader of a fleet to control. Each leader will be part of a fleet of leaders controlled by a main unit resulting in a relation tree. This approach is more scalable than the centralised architecture, but highly dependent on tree-top elements (Alur et al., 2001). Third, a decentralised or distributed architecture in which each robot is controlled independently, but making decisions according to the information shared by the other robots. This system is highly fault-tolerant, but less efficient to achieve a global goal. One commonly-used architecture is Alliance (Parker, 1998). Finally, a hybrid architecture combining multiple architectures, where the main computer manages the global goal and can influence small teams of robots. These teams are similar to a decentralised architecture, which allows for an optimised solution while providing a fault-tolerant system. An example of such an architecture is (Parker and Zhang, 2009).

In space, the implementation of MRS solutions had already been studied. The main challenge was the need for a high level of automation and reliable handling of the lunar conditions (Alfraheed and Al-Zaghameem, 2013) (Leitner, 2009) showed many use cases of MRS in space, but mostly focusing on satellites constellation. LUNARES (Cordes et al., 2011) presented a solution for heterogeneous multi-robot Moon exploration in which tasks were distributed from a ground station to a system of three heterogeneous robots. The variety of the robots allowed fulfilling a variety of missions linked to Moon exploration, similarly RIMRES was an extended approach that implied more sophisticated robots (German Research Center for Artificial Intelligence GmbH, 2022).

To this end, robotic missions on the Moon and Mars were based on single robots that did not interact or operate with other robots. As a result, their network architecture did not consider multiple robots in the same network, and their level of autonomy was limited despite their complexity. Future MRS will likely require a network architecture that allows multiple agents in the same network. Additionally, operating multiple robots requires coordination between the robots and a higher level of autonomy to handle this coordination efficiently. REALMS aims to address these issues for future lunar missions.

## 3 System description

*REALMS* offers to collaboratively address the challenges of a lunar exploration and prospecting mission.

### 3.1 Problem statement

ESA and ESRIC propose the ESA-ESRIC Space Resources Challenge to motivate the innovation for planetary prospecting technologies focused on the lunar environment. The objective consists of gathering visual data and generating a 3D map of an unknown environment with illumination and communication delays to be expected during a lunar mission. In the challenge stage, the illumination was set up in a dark hall with black curtains and an array of bright spotlights to replicate sunlight with a low incidence angle, similar to the lunar south pole. The communication delay is achieved by using the ESA delay communication system to simulate the delay between the Earth and the Moon at a software level. The round-trip delay consists of five seconds in total. Additionally, it is expected that the proposed system should be able to operate with occasional and eventual communication blackouts. The environment is a flat concrete surface with several obstacles*,* such as rocks and ramps. The goal is to reach a region of interest (ROI), representing a large crater, filled with small rocks on the soil. It contains larger rocks that need to be analysed by the research teams. The ROI can be accessed through a ramp.

Then, taking into account the challenge description, the following requirements are identified:

1. The system must map as much as possible of the 2,500 m^2^ area in 2.5 h.
2. The system must be able to move and explore a lunar surface analogue zone and navigate through rocks and slopes.
3. The system must be impervious to a five seconds delay, unpredictable blackouts and a limited bandwidth.
4. The system must be resilient to partial system failure, allowing to finish the mission even when parts of the system fail.

### 3.2 Proposed solution

**FIGURE 2:**
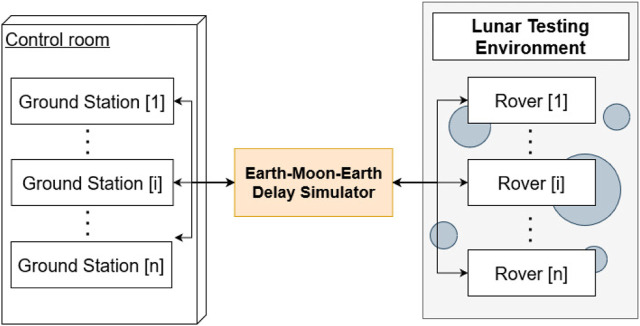
Overview of the REALMS architecture showing how multiple ground stations connect to multiple rovers through the Earth-Moon-Earth Delay Simulator.

The implemented system consists of two identical rovers controlled by two identical ground stations over a delayed network. This whole system can be extended to any number of rovers and ground stations, depending on the bandwidth available. This section explains the whole REALMS architecture composed by *n* rovers and ground stations, the Earth-Moon-Earth delay simulator and the lunar testing environment as shown in Figure 2. The control room with the ground stations to control the rovers is connected to the lunar testing environment through an Earth-Moon-Earth Delay Simulator. Inside the lunar testing environment, the rovers are connected through a wireless connection. The rovers can communicate to their respective ground station through the delay simulator, with a communication delay of 2.5 s in each direction of the data transmission.

**FIGURE 3:**
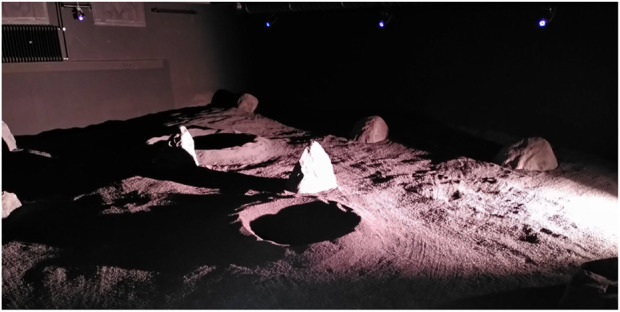
LunaLab, University of Luxembourg. This facility is equipped with an illumination system that resembles the lighting conditions of the lunar south pole.

This setup with the control room, the delay simulator, and the lunar testing environment is replicated in the LunaLab (Ludivig et al., 2020). The LunaLab is the lunar analogue facility of the University of Luxembourg, a 8 × 11 m^2^ room containing 20 tons of basalt focusing on the optical fidelity with respect to lunar environments (Figure 3).

The next sections elaborate on the different components in more detail. First, we describe the Earth-Moon-Earth delay simulator that adds communication delay in the network. Second, we explain the ground station setup. Third, we present the hardware and software components of the rovers.

#### 3.2.1 Earth-Moon-Earth Delay Simulator

**FIGURE 4:**
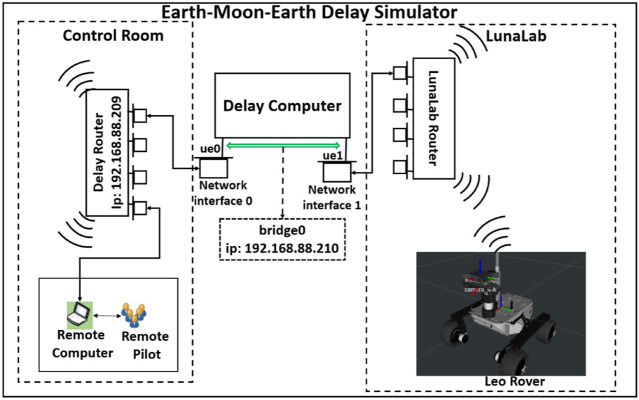
Delay Network Architecture connecting the rovers to the ground stations of REALMS by delaying all network traffic by a pre-defined amount of time.

Figure 4 describes the developed network architecture of the lunar delay network (Krueger, 2021) to test the performance of the proposed system. The system represented here connects the control room to a network in the lunar analogue facility by introducing a delay of 2.5 s in each direction of the connection. The delay computer has a 3.0 GHz Intel Core *i*7 generation 8 processor, and 8 GB of RAM. The operating system that we use is FreeBSD 12.2. The delay computer has two separate network interfaces, *ue*0 and *ue*1, as described in Figure 4. There are two routers, Delay Router and LunaLab Router, connected to *ue*0 and *ue*1, respectively. All the remote computers controlling the navigation and movement of the rovers are connected *via* Ethernet cable to the Delay Router. Also, the *REALMS* rovers are connected to LunaLab Router *via* 2.4 GHz Wi-Fi signal. In order to emulate an end-to-end delay between the remote computer and the rovers, there is a bridge, called *bridge*0, between *ue*0 and *ue*1. Therefore, all the traffic passes through the bridge between the control room and the LunaLab. Finally, two rules are set for the outgoing traffic from each network interface that is connoted to the bridge (*ue*0 and *ue*1) using the “ipfw” command to introduce the specific delay.

#### 3.2.2 Ground stations

**FIGURE 5:**
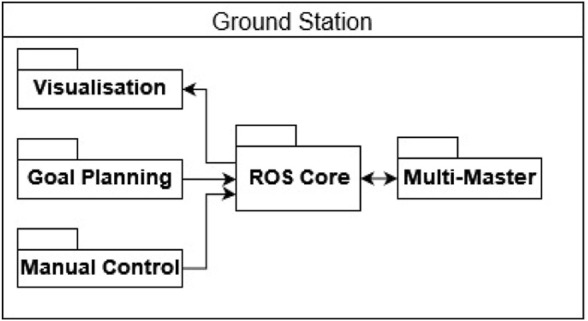
REALMS ground station architecture diagram showing commands sent to the robot and visualisation based on data received by the rover.

The rovers are controlled through computers that serve as a ground station. Figure 5 shows the functions of the ground stations. They include visualising the rovers and their environment, the possibility of giving navigation goals to the rovers and the ability to teleoperate them. The communication between the ground stations and the rovers is established through FKIE multimaster nodes. Each ground station is running a ROS Master.

RViz is used as a user interface and allows to send a navigation goal to the rovers. The FKIE multimaster software enables RViz to control the rovers despite the presence of network delay. Each robot is unaware of the other robots in the network allowing for easy scaling of the network and reducing interference between the robots. Additionally, the ground station can switch to manual mode for teleoperation of the robot *via* input devices.

#### 3.2.3 Rover

The rovers used for REALMS are two off-the-shelf robots modified according to the needs of lunar exploration and mapping in lab conditions. The following sections will present the hardware and software of the REALMS rovers.

##### 3.2.3.1 Hardware

**FIGURE 6:**
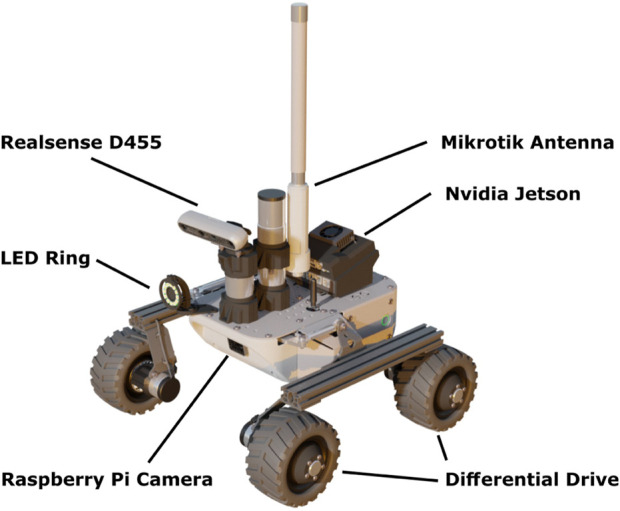
Overview of the REALMS Leo Rover hardware.

Each rover is a modified version of a Leo Rover (Kell Ideas, 2021). It has a mass of 6.5 kg and a footprint of 45 × 45 cm. The drive system is based on a differential drive mechanism where each wheel can turn independently. Figure 6 shows a Leo Rover used for REALMS with all the relevant components, such as the cameras, the communication antenna, the Nvidia Jetson, the lights and the wheels.

The rover is equipped with two different computers. The main embedded computer is a Raspberry Pi *v*4*B* using software provided by the Leo Rover manufacturer. This computer runs the ROS Master, the communication to the motor driver, the onboard illumination system, and a dedicated Raspberry Pi camera used for teleoperation. Additionally, the rover has LED rings composed of 12 SK6812-based LEDs. They can illuminate the surface in front of the rover and guarantee sufficient visibility of terrain features, addressing the mapping requirements. The Raspberry Pi is transferring commands to the motor driver board, a Core2-ROS designed specifically for the Leo Rover. On the other hand, the second computer is an *Nvidia* Jetson Xavier NX running in 15 W power mode. It executes the Real-Time Appearance Based Mapping (RTAB-Map) (Labbe and Michaud, 2019) vSLAM algorithm based on the images and point cloud captured by an RGB-Depth (RGB-D) camera and a path planner. It has sufficient computational power to reliably run the vSLAM software without delays in the mapping process while keeping low power consumption. The Nvidia Jetson Xavier allows to distribute the computational workload while adding redundancy to the system for increased resilience.

The RGB-D camera used for the vSLAM algorithm is an Intel RealSense D455 with an integrated Inertial Measurement Unit (IMU), which allows navigating in feature-poor environments. The camera uses a resolution of 1,280 × 720 pixels at a frame rate of 5 frames per second (fps). The RGB-D camera with IMU is the sole input for odometry. Wheel odometry is not used as it is considered unreliable for loose soil*,* as can be found on the lunar surface. The RealSense D455 camera is cost-effective, accurate at low ranges and computationally lightweight for the connected device. The camera stream of the RealSense D455 is only used locally for the vSLAM algorithm. The data is not sent to the ground station since this would generate high bandwidth demands.

The two embedded computers allow sharing the workload between them. The most computationally expensive programs run on the Jetson, leaving all the critical functionality, such as telecommunication and wheel control, to the Raspberry Pi. If the Jetson fails, the Raspberry Pi can still be used for teleoperation, providing additional reliability. As for the networking, the two computers are connected to a Mikrotik WLAN router through a network switch, connecting them to the external network.

**FIGURE 7:**
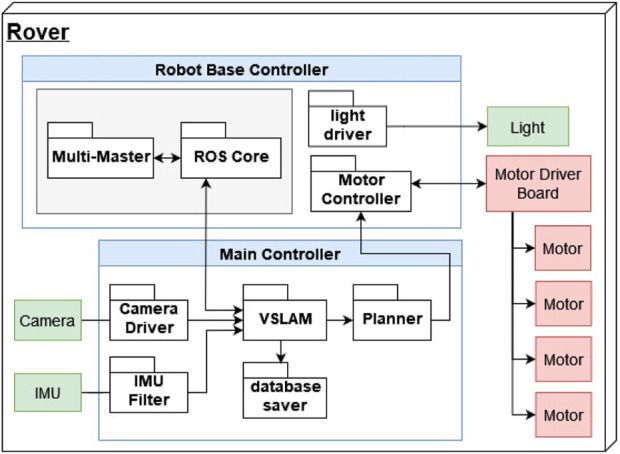
REALMS robot architecture diagram showing the robot base controller hosting the ROS Master, the hardware drivers and the main controller hosting the vSLAM system with the sensor input and the path planner.

The robot architecture of the system used in this work is presented in Figure 7. The Raspberry Pi is used as the robot base controller. It handles the motor controller and the lights of the rover. It also contains the ROS Master and the FKIE multimaster node. The Jetson is the main controller for higher-level software such as the vSLAM algorithm and the path planner. Additionally, it runs an IMU filter, the camera driver for the RealSense camera and a database saver that will copy the RTAB-Map database to the ground station.

The vSLAM algorithm uses the visual input of the RealSense camera and its IMU data after filtering it. It then reconstructs the terrain and saves this data in a database as a 3D point cloud and a 2D map. At the same time, this map serves as base input for the path planning algorithm together with the odometry information provided by the vSLAM node. The path planner generates the commands for the wheel drivers to move the robot to the planned position. The lights of the rovers are turned on and off through a call directly from the operator.

##### 3.2.3.2 vSLAM

This software component solves the first requirement to map the largest area possible inside the lunar environment. It allows REALMS to create a map of the environment and localisation of the rovers based on visual inputs only, avoiding drift induced by wheel slip (Yang et al., 2006), a common issue on lunar terrain. For the vSLAM*,* a modified version of RTAB-Map (Labbe and Michaud, 2019) is used. The input data are RGB-D images and data from an IMU. The default version of RTAB-Map generates false obstacles within the 2D local cost-map preventing the optimal navigation of the robot. The false obstacles originate from noise in the 3D point cloud that creates artefacts below the terrain. These are due to the natural reflection of the light on the ground which makes the depth acquisition by the RealSense noisier. To avoid this, the modified algorithm rejects points from the 3D point cloud below a threshold value in the *z*-axis while generating the 2D map. For this work, RTAB-Map provides the following advantages: It has good documentation on how to integrate it into ROS-based systems. It provides multiple interfaces for different camera types, allowing for flexibility in selecting the input sensors. It also supports multi-session mapping, a feature that enables REALMS to perform offline map merging through a command line interface.

##### 3.2.3.3 Path planner and follower

This component focuses on solving the second requirement to navigate inside the environment. It is in charge of producing the necessary manoeuvres to make the rover autonomously drive from one location to another. To do this, the planner calculates a path connecting the rover’s location to the target location as the initial step. The path planning algorithm used for REALMS is the Dynamic-Multi-Layered Path Planning (DyMu) (Sánchez-Ibánez et al., 2019) algorithm, which is developed by ESA. Thereafter, the planner dynamically generates manoeuvres to make the rover follow this path.

The path planning relies on the Fast Marching Method (FMM) (Kimmel and Sethian, 2001). This method numerically solves the propagation of a wave originating from the robot location. The wave expands over a cost map, consisting of a grid where each node has an associated cost value. Depending on this value, the wave expands more or less at the location of the corresponding node. After the wave propagation is calculated, a gradient descent method extracts the path from it. The generated path is optimal in the sense that it is the curve connecting the two locations of interest with the minimal amount of accumulated cost along its way. Each node has an assigned positive non-zero cost value in the grid, ensuring that the calculation of the wave propagation does not degenerate. Unlike other commonly used methods such as A* or D*, this path does not necessarily need to pass through the grid nodes, and hence its shape is not restricted to the grid topology. Path following is based on the Conservative Pursuit (Filip et al., 2017). An improved version of the Pure Pursuit algorithm ensures the rover is always close to the path within a specified threshold. Its performance was already tested in past field tests (Gerdes et al., 2020). The DyMu planner is used for REALMS as it is developed by ESA for rovers operating in unstructured terrain. The default path planner and other path planners, such as A* and D*, use a grid-based solution. Instead, the DyMu planner is not limited by the constraints of a grid and therefore is well suited for the use case of REALMS.

##### 3.2.3.4 Multimaster

The multimaster component focuses on overcoming potential issues with the communication delay and loss as well as increasing the resilience of the entire system, hence addressing the third and fourth requirements. It allows running one ROS Master on each system element and thus ensures that the topics are only shared between a ground station and its corresponding robot. The ROS Master is a central part of the ROS ecosystem as it handles topics, services and actions, registers which nodes are publishing and subscribing, holds the parameter server and directs the data traffic to the corresponding nodes. By conventional definition, there is only one single ROS Master in a given network of robots to handle all the ROS data traffic within the system. Multiple robots can share a single ROS Master, however this leads to a centralised architecture, more prone to failure, especially when the connection to the ROS Master gets interrupted.

We integrate the FKIE multimaster (Fraunhofer-Institut für Kommunikation, Informationsverarbeitung und Ergonomie FKIE, 2017) in REALMS to prevent communication issues between the ground station and the robots by connecting multiple ROS Master instances and sharing topics between them. It comprises two main components, discovery and sync. Discovery can show all the ROS Master instances available on a network. Sync is used to get the topics and messages from the desired ROS Master.

The two aforementioned components are set up to allow sharing only the correct rover’s topic with the desired ground station. This is done by using the option sync_hosts filled with the IP address of the robot and the ground station.

## 4 System analysis

Each requirement in subsection 3.1 is analysed and the system designed to meet them accordingly. Table 1 shows how each component addresses each requirement. A component can serve as a key component (K) or supportive component (S). A key component is responsible to meet one of the requirements, while a supportive component contributes partially to meet a requirement in a non-essential way.

**TABLE 1:** Components addressing the system requirements.

	Requirements			
**Components**	(K: Key Component, S: Supportive Component)			
	**Mapping**	**Movement**	**Delay**	**Resilience**
Lights	S	S		
Motors	S	K		
Camera	K	S		
IMU	S			
vSLAM	K			
Planner	K			
Multimaster			K	S
Multi robot	S	S		K
Visualisation	S	S		
Dual control mode	S	S	K	K

### 4.1 Mapping coverage

**FIGURE 8:**
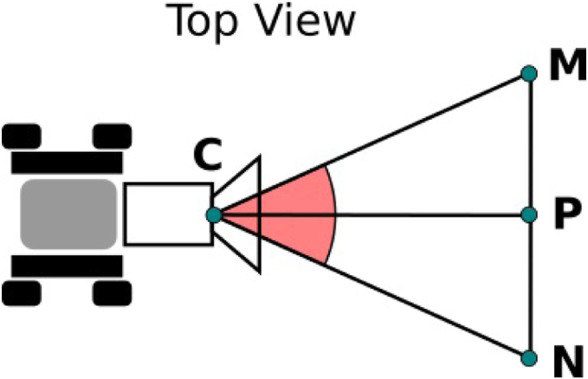
Top view schematic of the camera field of view. NM is the width of the field of view.

It is expected that the MRS must cover a large area and create an associated map in 3D within a limited time. In the case of the Space Resources Challenge, the explorable area is specified as 2,500 m^2^. The mapping is done with a theoretical maximum movement speed of 0.04 m/s while using the autonomous control by sending goals to the robot. The camera used by the rovers has a field of view allowing to map 4.6 m^2^, in the shape of a trapeze. As shown in Figure 8, when considering a triangle *CNM* representing the field of view of the camera, where *P* is in the centre of *NM*, the angle *∠ NCM* is equal to the horizontal field of view *FoV*_*H*_ of the camera and has a value of 87°. The distance *NM* is the width of the projected field of view on the ground surface that the robot can scan.

Z-distances in the camera frame larger than 3 m are assumed to be unreliable due to high noise, so *CP* is set to 3 m. The distance *NM* is then 5.69 m, according to (1):

$$NM=2\times CP\times \text{tan}\frac{FoV_{H}}{2}$$ (1)

Assuming each rover moves at an average speed *v* of 0.025 m/s in a straight line without encountering any obstacle, each rover can cover an area *a* of up to 1,281.1 m^2^ in 2.5 h, according to: *a* = *v* × *NM* × *t* where *t* = 2.5 *h* $\times 3600\frac{s}{h}=9000$ s. If two robots map simultaneously with a 20% overlap, they can cover an area *a*_*tot*_ of up to 2049.8 m^2^ in 2.5 h, according to (2):

$$a_{\mathrm{tot}}=2\times a\times 0.8$$ (2)

To verify the coverage, an experiment is carried out to measure the time necessary to cover the LunaLab at Centre for Security, Reliability and Trust (SnT) with a single robot. The laboratory has an area of 88 m^2^. Mapping the entire facility with a single robot takes on average 12 min 30 s, which is equal to 750 s. As a result, in 2.5 h, a single robot can cover up to $a_{e}=88\times \frac{2.5\;h\times 3600\frac{s}{h}}{750s}=1,056.0$ m^2^ where *a*_*e*_ is the estimated surface covered by a single rover during the challenge based on experimental values.

If two robots map simultaneously with a 20% overlap, they can cover an area *a*_*tot*_ of up to 1,689.0 m^2^ in 2.5 h, according to (3):

$$a_{\mathrm{tot}}=2\times a_{e}\times 0.8$$ (3)

Based on the mission requirements, the robots must explore an area of 2,500 m^2^. The estimated maximum area covered by two robots, determined numerically and experimentally, are below the target area specified in the requirements. As a result, the REALMS rovers can not map the target area within 2.5 h. However, the system is technically capable of covering more than 50% of the area defined in the requirements. Also, the system can be improved and scaled up in future works to cover larger areas.

### 4.2 Environment constraints

#### 4.2.1 Minimum clearance

**FIGURE 9:**
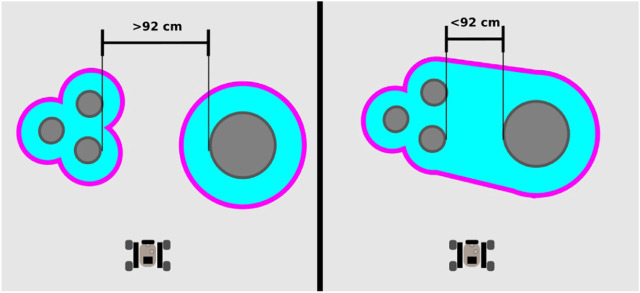
Graphical representation of the minimum clearance of the path planner. Objects closer than 92 cm are considered as too narrow for the planner to traverse in between those objects.

A rover needs to operate safely in an unknown terrain for lunar exploration. It needs to keep a safe distance from obstacles in the environment to prevent collisions that can damage the robot. At the same time, the rover needs to traverse between obstacles to access new areas to explore. This is a trade-off between safety and mobility. The path planner is configured to avoid entering into gaps narrower than 92 cm. This value is defined by the dimensions of the Leo Rovers plus a safety margin of 23.5 cm on each side. This is depicted in Figure 9. If necessary, the robots can be cautiously teleoperated through narrow spaces.

#### 4.2.2 Maximum slopes

**FIGURE 10:**
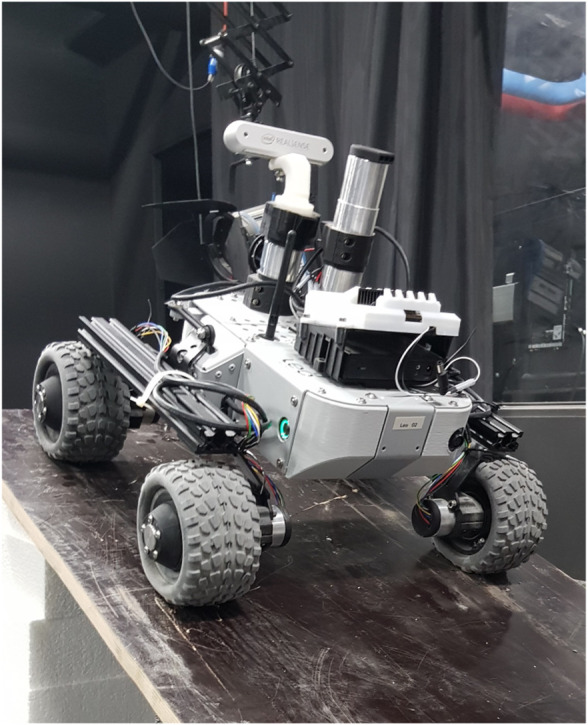
Experiment to determine maximum slope inclination the rovers can traverse.

In the permanently shadowed regions of the Moon, a robot needs to handle slopes of up to 22.1° (Gläser et al., 2018). We measure the maximum inclination angles the REALMS rovers can mount. They traverse a ramp as shown in Figure 10 multiple times using three different surface materials while gradually increasing the inclination angle. In this way, we discover the values of the maximum inclination angle the rovers can climb according to these materials. The maximum angle is 30° for loose basalt, 22.5° for a solid wooden surface and 26.6° for an aluminium surface. The friction on basalt is higher than on aluminium which causes the wheels to slip on aluminium.

### 4.3 Delay invariance

Standard software has a timeout function implemented. This function stops the program if no data is received in a certain amount of time. The timeout function prevents communication when there is a total communication delay of 5 s as is the case in Earth-Moon-Earth communication. The visualisation software RViz needs to connect to a ROS Master as otherwise, it returns an error after a timeout of 1 s. For terrestrial applications, it is common to run a single ROS Master in the robotic network where one robot contains the ROS Master, and the ground station is a slave connecting to the ROS Master of the robot. The communication delay does not allow this connection due to the timeout. REALMS overcomes this issue by running a ROS Master on each device involved. This way, RViz and similar software always receive inputs from their local ROS Master. The FKIE multimaster software is bridging the communication between the individual ROS Master instances, making the system delay invariant as it does not implement a timeout for the communication between the ROS Master.

### 4.4 Communication blackouts

The challenge contains periods with communication blackouts to represent scenarios where the communication antennas temporarily have no clear line of sight to transmit data. During a communication blackout, the rovers can move autonomously until they reach their goal and then wait for a new goal. While the communication is cut, the rovers keep waiting for new instructions, while the ground station sends the next commands to the rovers as soon as the connection is re-established. Due to the FKIE multimaster, communication with the ROS Master is ensured for the robots and the ground station. The timeout functions of the ROS nodes are not triggered since the communication to the local ROS Master is still intact. The re-establishment of the communication between the rovers and the ground station is handled by the FKIE multimaster and its discovery function that allows connecting to an existing ROS Master.

Any packages lost during the communication loss are not retrieved. The rovers process the sensor data onboard and only send mapping data, odometry information and a low-resolution greyscale video stream back to the ground station. The only data sent from the ground station are either position goals for autonomous navigation, direct teleoperation commands or signals to trigger minor actions such as turning on or off the lights or partially restarting the system. In case of lost data packages, the commanded action must be repeated.

### 4.5 Resilience

The resilience of a system is its ability to recover after a partial failure. In the case of this challenge, it is important to see if all the previous requirements can be matched even with a faulty component. REALMS consists of a defined number of rover-ground station pairs. The bandwidth limits the maximum number of pairs. The ROS Master running on each machine make the system more robust as each robot and its corresponding ground station are not interdependent. If one of the two members is faulty, it can still be used to operate another member.

The REALMS used for the challenge is composed of two rover-ground station pairs, reducing the risk of failure by adding redundancy in the system architecture. Having more than one pair assures resilience and higher tolerance to potential blackouts. Additionally, the use of the multimaster setup makes the system resilient towards communication delay. The maps created by each robot are saved locally. Each map can be retrieved by the ground stations and merged on the ground stations, allowing to use an incomplete map to enhance the global map. At this point, the REALMS rovers are ready to face the lunar surface like environmental conditions expected in the challenge.

### 4.6 Mission control

**FIGURE 11:**
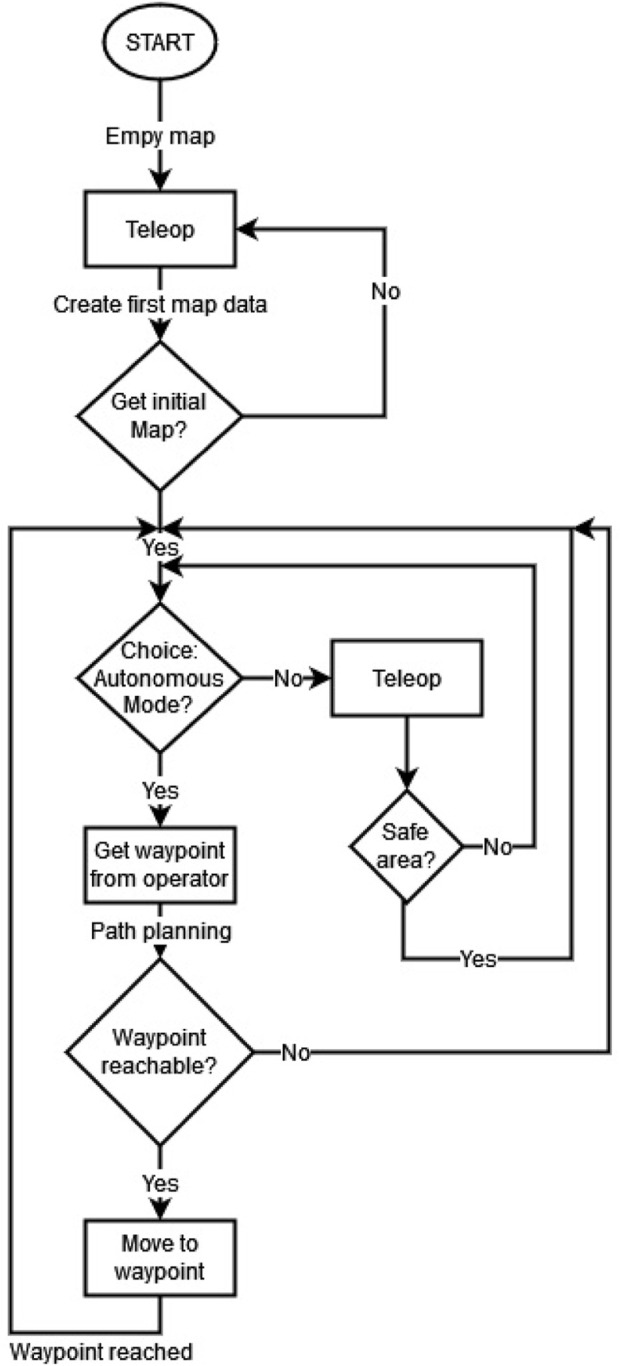
Work flow of the semi-autonomous approach based on receiving waypoints through a human operator and allowing teleoperation.

REALMS is designed to map an unknown environment with multiple rovers in a semi-autonomous approach defined by a human-in-the-loop system. A human operator can provide waypoints to the system and the rovers can reach these waypoints autonomously, provided the path planner can find a feasible path. Otherwise, the human operator can take control and teleoperate the robot to cross difficult areas, such as spaces too narrow for the robot to safely navigate autonomously. Figure 11 shows how the robot is controlled by first using teleoperation until the robot creates the first frame of a map. After this initialisation, the operator can switch to the autonomous mode or keep teleoperating the robots. In the event that the robot cannot plan a path to a given waypoint, the operator can choose a new waypoint or drive manually until it is safe to revert to autonomous mode.

During this entire process, the RTAB-Map database is copied to the ground station as a background task. The database can then be used to keep a safe copy of the 3D point cloud throughout the challenge while also allowing to merge the 3D point clouds of the two rovers offline at the end of the challenge to have a full overview of the mapped area.

## 5 Experimental results

### 5.1 Testing REALMS in the LunaLab

To test the multi-robot mapping capabilities of REALMS, the two rovers are placed in two different locations inside the LunaLab.

**FIGURE 12:**
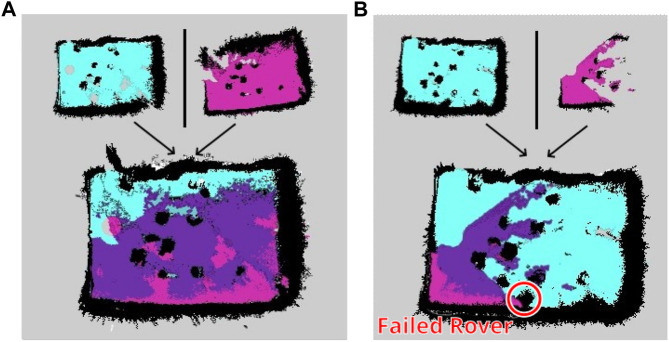
Mapping of the LunaLab done by two REALMS rovers in two different cases. **(A)** Two rovers successfully *map* a shared environment and *merge* their maps. **(B)** Two rovers mapping a shared environment with one rover failing in the process and the other taking over the area.

Two scenarios are tested. Scenario one shows the successful mapping of a shared area with two robots (Figure 12A). The light-blue map is made by the first rover, mapping the top side of the LunaLab, while the pink map shows the part mapped solely by the second robot on the bottom side of the lab. In the bottom map, the purple area shows the overlapping part that is mapped by both robots. The entire experiment is realised in 6 min and 48 s. The second scenario simulates the case of a system failure on one of the two rovers where the other rover can cover the missing area so that the mapping can be executed with some coverage limitations or requiring more time to cover the remaining area. In the test case, the second rover is simply shut down to remain unresponsive. The first rover perceives it as an obstacle on the map. Possible causes for a rover to fail are low battery charge, one or more wheels getting stuck in the loose soil or loss of odometry. In all of these cases, the robot stops moving. If the cause is loss of signal, the robot also stops after not receiving new instructions to move while in teleoperation mode, and it pursues to reach its goal in autonomy mode. Figure 12B shows a scenario where the second robot experiences an issue after 1 min 30 s and is unable to continue. The position of the failed rover is highlighted with a red circle. The first rover can cover the remaining area resulting in less overlap. As a result, the total laboratory area is still covered even in the event of a partial system failure. This experiment takes 9 min 2 s.

### 5.2 Using REALMS during the ESA-ESRIC Space Resources Challenge

The validation experiment of REALMS is the first trial of the ESA-ESRIC Space Resources Challenge. This trial consist of 6 h of preparation and 2.5 h to realise the mission. The mission takes place in an area of 34 × 47 m^2^. Two-thirds of the area have a concrete surface, while the last part, the ROI, is made of small rocks of 3–5 cm diameter. The ROI represents the inside of a crater with a rim made out of piled-up rocks. A ramp across the rim allows the rovers to access the ROI. The first area is filled with rocks, creating a path across two more ramps that lead towards the ROI. These obstacles force larger robots to follow a precise path, passing across the ramps and covering most of the area.

**FIGURE 13:**
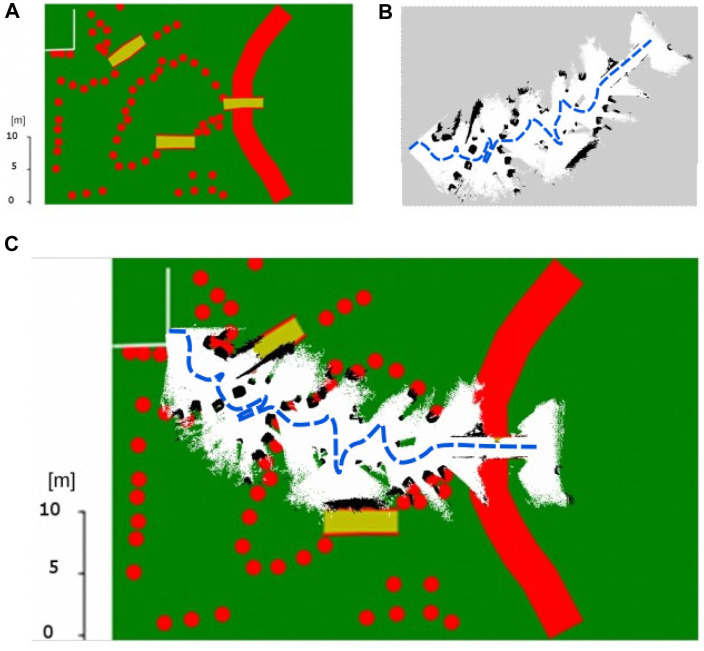
**(A)** Map provided by ESA at the beginning of the mission. **(B)** Map created by one rover during the mission. **(C)** Map of the lunar environment of the challenge overlaid with the map generated by the REALMS rover. The two maps are matching, showing the solution is accurate.

At the beginning of the challenge, the robots are placed in the starting area. Meanwhile, the operators are in a control room with no contact with the outside. In the control room, a network is available to connect to the rovers while adding a delay to the communications with the robots. A hand-drawn map of the lunar area is provided to the operators, giving a general idea of the zones to explore. Figure 13 shows the map handed out to the operators, with the generated map by REALMS overlaid on top of this map.

### 5.3 Results of the ESA-ESRIC Space Resources Challenge

Despite several communication blackouts, the mission is completed successfully as one rover reached the ROI within the time frame of 2.5 h. The remaining rover moves in between the large rocks and moves straight to the ROI. The second rover is meant to follow the predefined path and increase the map coverage. Unfortunately, the second rover is lost after a communication blackout at the beginning of the mission, leaving the rover unresponsive to commands. A possible reason for this might have been the limited bandwidth of the network. The first rover sends a low-resolution video stream and the map to the ground station, possibly using too much bandwidth so that the discovery function of the second rover cannot receive the discovery messages of the FKIE multimaster node. Figure 13 shows the area that the first rover has mapped during its traverse to the ROI. The traverse path is added in a blue dotted line. It shows that the rover has passed in between the obstacles and avoided the ramps. This is a strategic plan to bring the first rover to the ROI as quickly as possible while the second rover maps larger parts of the environment. Since the second rover loses connection after a communication loss, the second rover had no contribution to the mapping. As shown in Figure 13, the ramps in the mission area are clearly represented in the map and also the obstacles in the mission area are mapped as in the provided map. REALMS can map some smaller obstacles, close to the last ramp, that are not included in the provided map. The vSLAM algorithm allows to keep track of the odometry during the entire mission.

**FIGURE 14:**
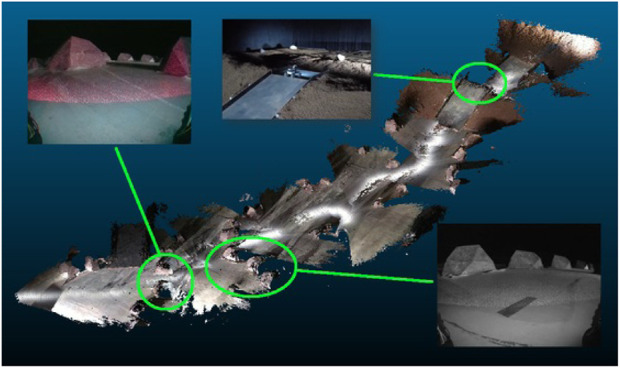
3D point cloud of the lunar environment of the challenge.

Uplinking the RTAB-Map database has too high bandwidth constrains and is not successful during the challenge. Therefore, at the end of the mission, the 3D point cloud generated by the vSLAM algorithm is retrieved from the rover. The 3D point cloud is represented in Figure 14. The rocks defining the path can be easily recognised in the 3D point cloud as well as the ramp leading to the ROI. Only the descending part of the final ramp is not represented correctly.

The final coverage achieved by REALMS was 310 m^2^. Based on our measurements, the entire challenge area is 1,598 m^2^, REALMS explores 19.4% of the total area. As only one robot was operational and considering the connection outages, it is necessary to avoid any further risks and drive more carefully. As a result, the system achieves 24.2% of its experimental capability, which is an encouraging result. This system is selected among 13 teams to continue the challenge and serves as a base for the final trial.

## 6 Discussion and lessons learnt

Participating in this challenge teaches us valuable lessons regarding the deployment and use of MRSs in extreme environments. In the following, we present a list of lessons learned during the ESA-ESRIC Space Resources Challenge.

1. In ROS, a robotic system has a single ROS Master by default. With the communication delay between the lunar surface and the ground station, the ROS Master on the robot can not be found by some nodes launched on the ground station. This includes RViz for visualisation and controlling the robot due to a software timeout. Additionally, having two rovers in the field at the same time requires that one ROS Master will handle two robots. The FKIE multimaster package makes it possible to connect multiple ROS Masters in a single robotic system. This allows the robots to have a ROS Master on each robot and one on each computer of the ground station, avoiding the software timeout and increasing the independence of the two rovers.
2. The Leo Rovers are not initially designed to use namespaces for their nodes, topics, robot model links and joints. As a result, one robot responds to the other robot’s commands. This is resolved by isolating the two ROS Master through the FKIE multimaster package. Hence, it is reconfigured such that the robots would only listen to their corresponding ground station computer.
3. The default version of RTAB-Map causes noise. This shows that off-the-shelf components for terrestrial applications are limited when used in extreme environments such as the lunar surface. By customising the code, the mapping results can be improved.

Despite all these lessons, there are still several challenges that need yet to be addressed:

1. The communication architecture based on ROS 1 using the FKIE multimaster package does not provide the necessary stability to reliably connect to the robots.
2. The inter-robot communication is entirely depending on the provided access point during the challenge. This approach is less reliable and can increase network latency.
3. The resilience of the system is a major contribution to finishing the mission to this extent, given that one robot loses the connection to the ground station, the other rover can still operate.
4. The user interface easily scales on a system level, but not on a user experience level. Managing multiple robots on multiple operator computers is not feasible for large scale systems.
5. The bandwidth is limited to 100 Mbit/s which causes communication losses when engaging high data traffic, hindering the transfer of data towards the ground station.

## 7 Conclusion and future works

Exploring the lunar surface is a difficult task for a single robotic system. REALMS presents a system to increase resilience and coverage for robotic mapping tasks. This is achieved by using multiple small rovers that can work in parallel to overcome challenges like partial system failures and lead the mission to success. The possibility to grow the fleet size with additional rovers allows to increase the mapping capability and system resilience. The system shows its ability to perform during the Space Resources Challenge. It demonstrates the interest in a resilient system designed for lunar exploration.

Future works will take into consideration the lessons learned from the Space Resources Challenge. A major focus point will be the communication structure between the robots with respect of state-of-the-art decentralised network architectures. Such an architecture might increase the overall resilience of the system together with additional robotic agents and sensors used for vSLAM. ROS2 can provide an interesting solution as it is built with MRS in mind and allows connecting multiple robots avoiding the limitation to a single ROS Master per system without the need for external packages. Next, the user interface to control multiple robots will be adjusted to simplify the workflow and ease scalability. Lastly, the soil of the first trial of the ESA-ESRIC Space Resources Challenge as well as the basalt in the LunaLab do not consist of lunar regolith simulant. In future research, the use of regolith simulant will lead to more accurate representations of the lunar surface.

## Data Availability

The data supporting the conclusion of this article will be made available by the authors upon request. Only limited amounts of data are available due to constrains during the challenge.
